# 2-Methyl-4-thio­cyanato­aniline

**DOI:** 10.1107/S2414314625002160

**Published:** 2025-03-14

**Authors:** Erik de Lange, Eric Cyriel Hosten, Richard Betz

**Affiliations:** a Nelson Mandela University, Summerstrand Campus, Department of Chemistry, University Way, Summerstrand, PO Box 77000, Port Elizabeth, 6031, South Africa; Goethe-Universität Frankfurt, Germany

**Keywords:** crystal structure, rhodanided derivative, hydrogen bonds

## Abstract

The title compound, C_8_H_8_N_2_S, is a rhodanided derivative of *ortho*-toluidine. Classical hydrogen bonds of the N—H⋯N type, as well as C—H⋯N contacts, connect mol­ecules of the title compound into a three-dimensional network in the crystal structure.

## Abstract 2

The title compound is a rhodanided derivative of *ortho*-toluidine. The mol­ecules are connected into a three-dimensional network in the crystal structure by means of classical hydrogen bonds and C–H⋯N contacts.

## Graphical abstract

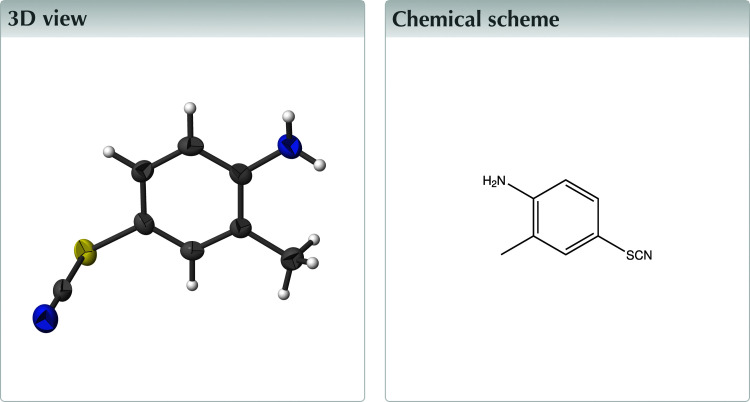

## Structure description

Aniline and its derivatives are valuable starting materials in synthetic organic chemistry and have found ample use in industrial processes, as is apparent in the historic establishment of the artificial dye and, subsequently, pharmaceutical industry (Griess, 1879 ▸; Bopp *et al.*, 1891 ▸). As an activated aromatic system, a large number of reactions is available for further functionalization of the phenyl group as well as the *ipso*-substitution of the amine functionality itself (Becker *et al.*, 2000 ▸; Sandmeyer, 1884 ▸), which allows for tailoring the physicochemical and spectroscopic properties of the target mol­ecules over a seemingly endless range. One particularly intriguing substituent on a phenyl moiety is the rhodanide (thio­cyanate) group as its cumulated double-bonding system allows for a number of fundamental follow-up reactions. In a continuation of our ongoing inter­est in structural aspects of aromatic amines such as halogenated anilines (Betz & Klüfers, 2008 ▸; Betz, 2015 ▸; Hosten & Betz, 2021*a* ▸,*b* ▸), anilines bearing protic (Betz & Gerber, 2011 ▸; Betz, Klüfers & Mayer, 2008 ▸; Betz *et al.*, 2011*a* ▸) or sulfur-based (Betz *et al.*, 2011*b* ▸) or organic substituents (Islor *et al.*, 2013 ▸; Hosten & Betz, 2021*c* ▸), we set out to explore the influence of a rhodanide group attached to the aromatic core of an aniline derivative. Structural information about organic thio­cyanates is still comparatively scant, however, the mol­ecular and crystal structures have been published for derivatives bearing the SF_5_ group (Okazaki *et al.*, 2014 ▸), an acetyl substituent (Kalaramna & Goswami, 2021 ▸), azo functionalities (Kakati & Chaudhuri, 1968 ▸; Aldoshin *et al.*, 1977 ▸; Sanjib *et al.*, 2004 ▸) or several meth­oxy groups (Ghosh *et al.*, 2019 ▸). Most intriguing in connection with our present study is structural information about two aniline derivatives bearing a thio­cyanate group (See & Zhao, 2018 ▸; Isakov *et al.*, 1977 ▸). Slightly more structural information is apparent for aromatic iso­thio­cyanated compounds such as, *e.g.*, the family of tri­fluoro­methyl benzene derivatives (Hasija *et al.*, 2023 ▸; Mandal *et al.*, 2023 ▸).

**Figure 1:**
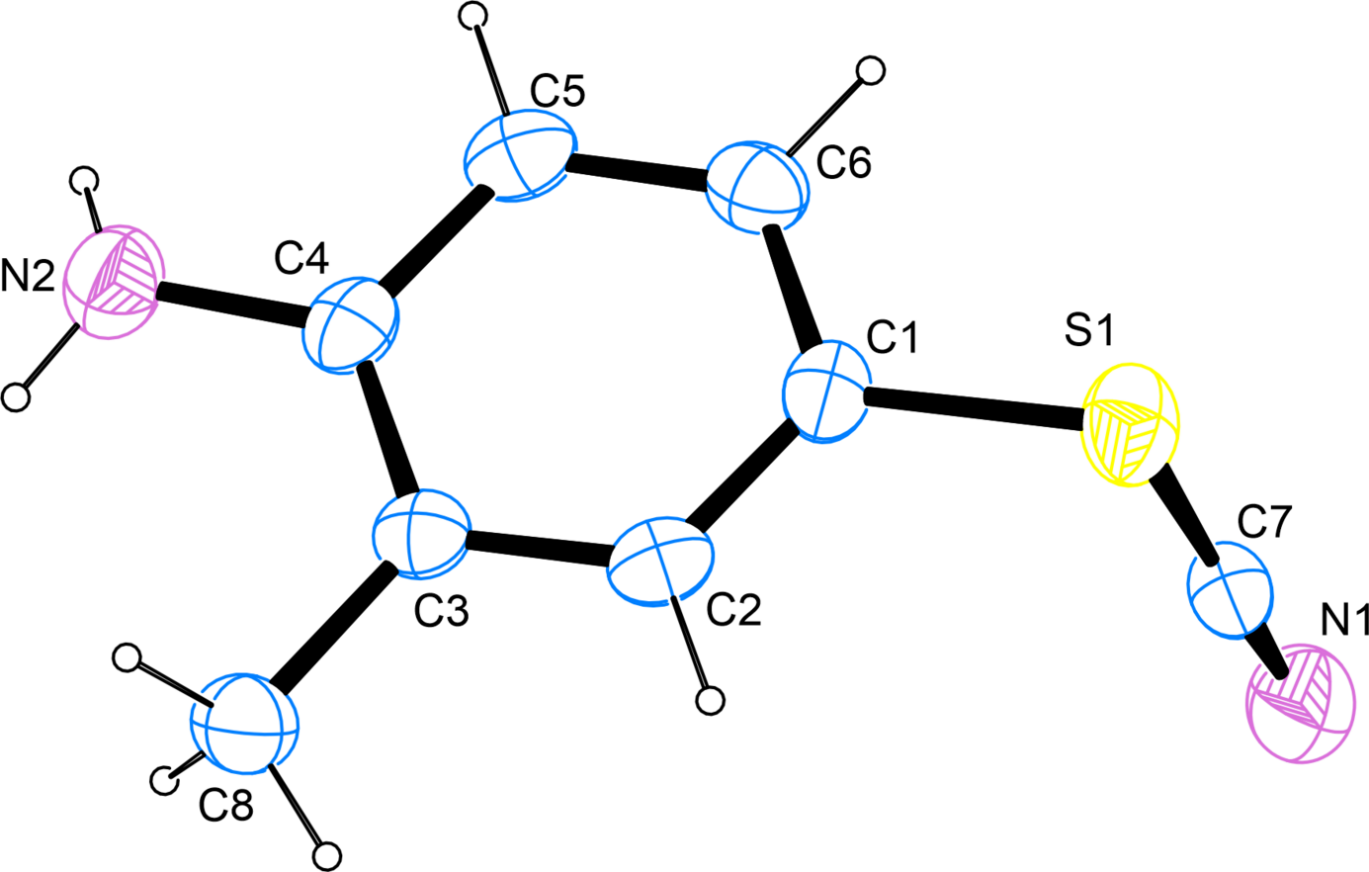
The mol­ecular structure of the title compound, with atom labels and anisotropic displacement ellipsoids (drawn at the 50% probability level).

The title compound is a derivative of *ortho*-toluidine bearing a rhodanide group in *para*-position to the amino group. The latter is bonded to the phenyl moiety *via* its sulfur atom. The thio­cyanate group is tilted out of plane of the aromatic moiety to an almost perpendicular position with the pertaining C7—S1—C1—C2 torsion angle measuring 86.6 (2)°. The C—S bond length of 1.692 (3) Å is in good agreement with other pertaining bond lengths in aromatic thio­cyanates whose mol­ecular and crystal structure have been determined on grounds of diffraction studies on single crystals and whose metrical parameters have been deposited with the Cambridge Structural Database (Allen, 2002 ▸). Intra­cyclic C—C—C angles span a narrow range of only 118.4 (2)–121.6 (2)° with the smallest angle found on the carbon atom bearing the methyl group and the largest angle on the carbon atom in between the carbon atoms bearing the rhodanide and the methyl group, respectively (Fig. 1 ▸).

**Figure 2:**
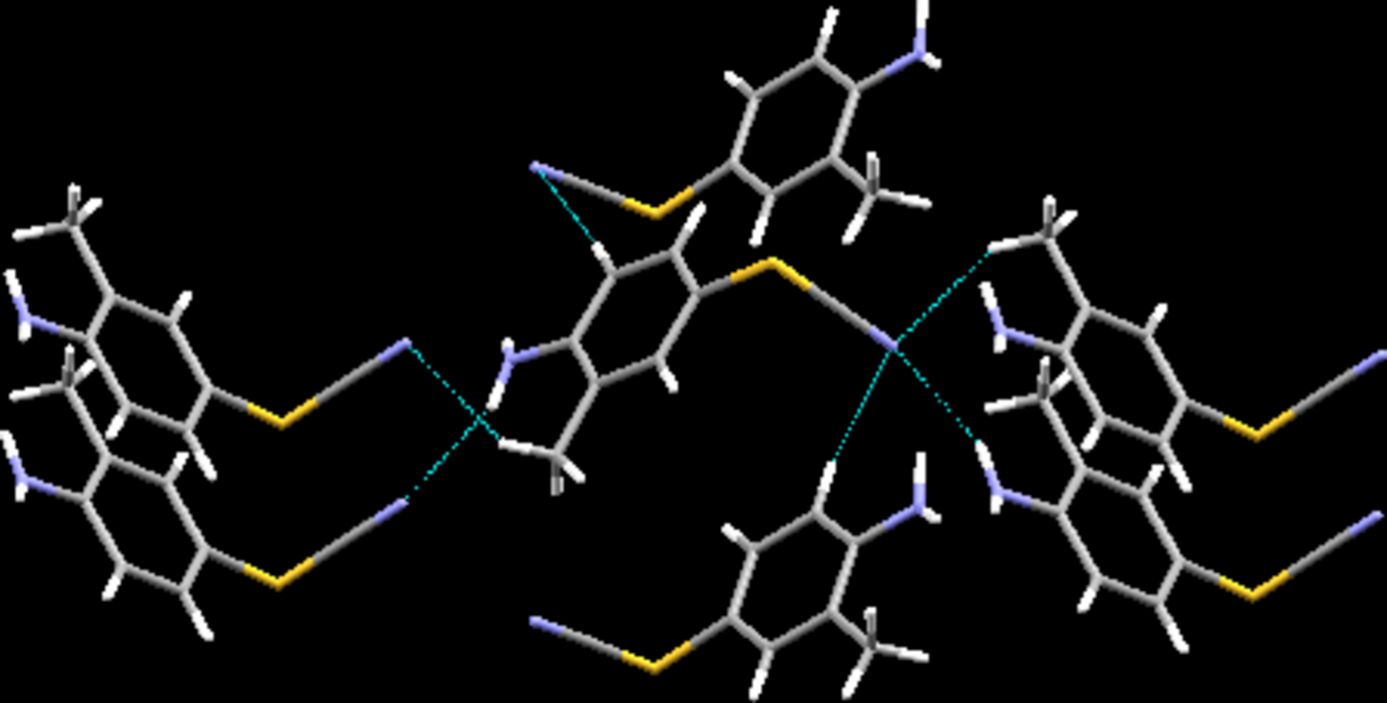
Inter­molecular contacts, viewed approximately along [110].

In the crystal, classical hydrogen bonds of the N—H⋯N type are observed next to C—H⋯N contacts (Table 1 ▸) whose range falls by more than 0.1 Å below the sum of van der Waals radii of the atoms participating in them. While the classical hydrogen bonds are established only by one of the two hydrogen atoms of the amino functionality as donor and the SCN group nitro­gen atom as acceptor, the C—H⋯N contacts are supported by one of the hydrogen atoms of the methyl group as well as the hydrogen atom on the carbon atom next to the amino group. The acceptor nitro­gen for the latter type of inter­actions is, invariably, the nitro­gen atom of the rhodanide group, thus denoting the latter atom as a threefold acceptor. In terms of graph-set analysis (Etter *et al.*, 1990 ▸; Bernstein *et al.*, 1995 ▸), the descriptor for the classical hydrogen bonds is 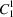
(9) on the unary level while the C—H⋯N contacts necessitate a 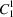
(7) 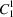
(8) on the same level. Overall, these inter­actions connect the mol­ecules to a three-dimensional network in the crystal structure. While π-stacking is not a prominent feature in the crystal structure of the title compound as the shortest distance between two centers of gravity was measured at 4.4380 (16) Å, it is worthwhile pointing out the short distance between the π systems as well as the sulfur atoms in neighbouring mol­ecules as the S⋯*C_g_* distance of only about 3.43 Å is comparable to the range that has been debated in the literature as an energetic minimum for the system benzene–hydrogen sulfide as well as in connection with pertaining metrical data obtained from the Protein Data Bank (Ringer *et al.*, 2007 ▸) (Fig. 2 ▸).

## Synthesis and crystallization

The compound was obtained following a standard procedure by reacting *ortho*-toluidine with KSCN and bromine in acetic acid (Becker *et al.*, 2000 ▸). Crystals suitable for the diffraction study were obtained upon free evaporation of the reaction mixture after workup at room temperature.

## Refinement

Crystal data, data collection and structure refinement details are summarized in Table 2 ▸.

## Supplementary Material

Crystal structure: contains datablock(s) I. DOI: 10.1107/S2414314625002160/bt4165sup1.cif

Structure factors: contains datablock(s) I. DOI: 10.1107/S2414314625002160/bt4165Isup2.hkl

Supporting information file. DOI: 10.1107/S2414314625002160/bt4165Isup3.cml

CCDC reference: 2429624

Additional supporting information:  crystallographic information; 3D view; checkCIF report

## Figures and Tables

**Table 1:** Hydrogen-bond geometry (Å, °)

*D*—H⋯*A*	*D*—H	H⋯*A*	*D*⋯*A*	*D*—H⋯*A*
C8—H8*A*⋯N1^i^	0.98	2.61	3.482 (4)	148
C5—H5⋯N1^ii^	0.95	2.63	3.576 (3)	172
N2—H2*B*⋯N1^iii^	0.85 (4)	2.38 (4)	3.185 (4)	157 (3)

Symmetry codes: (i) 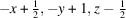; (ii) 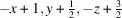; (iii) 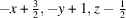.

**Table 2:** Experimental details

Crystal data	
Chemical formula	C_8_H_8_N_2_S
*M*_r_	164.22
Crystal system, space group	Orthorhombic, *P*2_1_2_1_2_1_
Temperature (K)	200
*a*, *b*, *c* (Å)	4.4380 (2), 10.5115 (4), 17.3105 (6)
*V* (Å^3^)	807.54 (6)
*Z*	4
Radiation type	Mo *K*α
μ (mm^−1^)	0.33
Crystal size (mm)	0.52 × 0.20 × 0.20

Data collection	
Diffractometer	Bruker APEXII CCD
Absorption correction	Multi-scan (*SADABS*; Krause *et al.*, 2015 ▸)
*T*_min_, *T*_max_	0.673, 0.746
No. of measured, independent and observed [*I* > 2σ(*I*)] reflections	26844, 1996, 1757
*R*_int_	0.062
(sin θ/λ)_max_ (Å^−1^)	0.667

Refinement	
*R*[*F*^2^ > 2σ(*F*^2^)], *wR*(*F*^2^), *S*	0.043, 0.075, 1.16
No. of reflections	1996
No. of parameters	109
H-atom treatment	H atoms treated by a mixture of independent and constrained refinement
Δρ_max_, Δρ_min_ (e Å^−3^)	0.26, −0.23
Absolute structure	Flack *x* determined using 628 quotients [(*I*^+^)−(*I*^−^)]/[(*I*^+^)+(*I*^−^)] (Parsons *et al.*, 2013 ▸)
Absolute structure parameter	0.00 (4)

Computer programs: *APEX2* and *SAINT* (Bruker, 2014 ▸), *SHELXS97* (Sheldrick 2008 ▸), *ORTEP-3* (Farrugia, 2012 ▸), *Mercury* (Macrae *et al.*, 2020 ▸), *SHELXL2019/3* (Sheldrick, 2015 ▸) and *PLATON* (Spek, 2020 ▸).

## full crystallographic data

### 2-Methyl-4-thiocyanatoaniline Crystal data

**Table:**

C_8_H_8_N_2_S	*D*_x_ = 1.351 Mg m^−^^3^
*M**_r_* = 164.22	Mo *K*α radiation, λ = 0.71073 Å
Orthorhombic, *P*2_1_2_1_2_1_	Cell parameters from 9369 reflections
*a* = 4.4380 (2) Å	θ = 2.3–28.2°
*b* = 10.5115 (4) Å	µ = 0.33 mm^−^^1^
*c* = 17.3105 (6) Å	*T* = 200 K
*V* = 807.54 (6) Å^3^	Rod, brown
*Z* = 4	0.52 × 0.20 × 0.20 mm
*F*(000) = 344	

### 2-Methyl-4-thiocyanatoaniline Data collection

**Table:**

Bruker APEXII CCD diffractometer	1757 reflections with *I* > 2σ(*I*)
φ and ω scans	*R*_int_ = 0.062
Absorption correction: multi-scan (SADABS; Krause *et al.*, 2015)	θ_max_ = 28.3°, θ_min_ = 2.3°
*T*_min_ = 0.673, *T*_max_ = 0.746	*h* = −5→5
26844 measured reflections	*k* = −12→14
1996 independent reflections	*l* = −23→21

### 2-Methyl-4-thiocyanatoaniline Refinement

**Table:**

Refinement on *F*^2^	Secondary atom site location: difference Fourier map
Least-squares matrix: full	Hydrogen site location: mixed
*R*[*F*^2^ > 2σ(*F*^2^)] = 0.043	H atoms treated by a mixture of independent and constrained refinement
*wR*(*F*^2^) = 0.075	*w* = 1/[σ^2^(*F*_o_^2^) + (0.0132*P*)^2^ + 0.3653*P*] where *P* = (*F*_o_^2^ + 2*F*_c_^2^)/3
*S* = 1.16	(Δ/σ)_max_ < 0.001
1996 reflections	Δρ_max_ = 0.26 e Å^−^^3^
109 parameters	Δρ_min_ = −0.22 e Å^−^^3^
0 restraints	Absolute structure: Flack *x* determined using 628 quotients [(*I*^+^)-(*I*^-^)]/[(*I*^+^)+(*I*^-^)] (Parsons *et al.*, 2013)
Primary atom site location: structure-invariant direct methods	Absolute structure parameter: 0.00 (4)

### 2-Methyl-4-thiocyanatoaniline Special details

**Table:**

Geometry. All esds (except the esd in the dihedral angle between two l.s. planes) are estimated using the full covariance matrix. The cell esds are taken into account individually in the estimation of esds in distances, angles and torsion angles; correlations between esds in cell parameters are only used when they are defined by crystal symmetry. An approximate (isotropic) treatment of cell esds is used for estimating esds involving l.s. planes.
Refinement. The carbon-bound aromatic H atoms were placed in calculated positions (C–H 0.95 Å) and were included in the refinement in the riding model approximation, with *U*(H) set to 1.2*U*_eq_(C).The H atoms of the methyl group were allowed to rotate with a fixed angle around the C–C bond to best fit the experimental electron density [HFIX 137 in the *SHELX* program suite (Sheldrick, 2015)], with *U*(H) set to 1.5*U*_eq_(C).Both nitrogen-bound H atoms were located on a difference Fourier map and refined freely.

### 2-Methyl-4-thiocyanatoaniline Fractional atomic coordinates and isotropic or equivalent isotropic displacement parameters (Å^2^)

**Table:**

	*x*	*y*	*z*	*U*_iso_*/*U*_eq_	
S1	−0.06454 (16)	0.57399 (7)	0.80755 (4)	0.0388 (2)	
N1	0.3355 (6)	0.5337 (2)	0.93268 (14)	0.0486 (7)	
N2	0.7653 (6)	0.6435 (3)	0.54134 (14)	0.0391 (6)	
C1	0.1997 (6)	0.5930 (3)	0.73078 (13)	0.0286 (6)	
C2	0.3097 (5)	0.4881 (2)	0.69152 (15)	0.0282 (5)	
H2	0.250656	0.405369	0.707623	0.034*	
C3	0.5039 (6)	0.5008 (2)	0.62927 (13)	0.0274 (6)	
C4	0.5861 (6)	0.6245 (2)	0.60565 (13)	0.0286 (5)	
C5	0.4736 (7)	0.7295 (2)	0.64576 (15)	0.0336 (6)	
H5	0.530092	0.812751	0.630098	0.040*	
C6	0.2820 (6)	0.7142 (2)	0.70775 (15)	0.0317 (6)	
H6	0.206848	0.786480	0.734497	0.038*	
C7	0.1766 (7)	0.5506 (3)	0.88190 (15)	0.0325 (6)	
C8	0.6234 (7)	0.3849 (2)	0.58817 (15)	0.0379 (7)	
H8A	0.557026	0.386128	0.534182	0.057*	
H8B	0.546710	0.308023	0.613493	0.057*	
H8C	0.844074	0.385071	0.590133	0.057*	
H2A	0.863 (7)	0.717 (3)	0.5391 (15)	0.035 (8)*	
H2B	0.871 (8)	0.582 (3)	0.5241 (18)	0.056 (11)*	

### 2-Methyl-4-thiocyanatoaniline Atomic displacement parameters (Å^2^)

**Table:**

	*U*^11^	*U*^22^	*U*^33^	*U*^12^	*U*^13^	*U*^23^
S1	0.0252 (3)	0.0540 (4)	0.0371 (3)	−0.0004 (4)	0.0033 (3)	0.0012 (3)
N1	0.0567 (18)	0.0541 (17)	0.0351 (13)	0.0112 (13)	0.0007 (13)	0.0003 (12)
N2	0.0423 (16)	0.0395 (15)	0.0354 (13)	−0.0051 (14)	0.0050 (12)	0.0018 (12)
C1	0.0215 (12)	0.0395 (15)	0.0248 (11)	0.0005 (12)	−0.0015 (10)	0.0024 (11)
C2	0.0255 (12)	0.0279 (12)	0.0312 (12)	−0.0029 (10)	−0.0081 (12)	0.0039 (11)
C3	0.0271 (15)	0.0290 (13)	0.0260 (12)	0.0010 (11)	−0.0064 (10)	0.0010 (10)
C4	0.0266 (13)	0.0355 (13)	0.0237 (11)	−0.0002 (12)	−0.0063 (11)	0.0038 (10)
C5	0.0386 (17)	0.0264 (13)	0.0359 (14)	−0.0046 (12)	−0.0050 (13)	0.0048 (11)
C6	0.0316 (14)	0.0302 (13)	0.0334 (14)	0.0045 (12)	−0.0046 (12)	−0.0018 (11)
C7	0.0358 (15)	0.0305 (14)	0.0312 (13)	0.0041 (12)	0.0092 (12)	−0.0012 (11)
C8	0.0453 (18)	0.0353 (15)	0.0331 (14)	0.0059 (13)	−0.0039 (13)	−0.0021 (11)

### 2-Methyl-4-thiocyanatoaniline Geometric parameters (Å, º)

**Table:**

S1—C7	1.692 (3)	C3—C4	1.411 (3)
S1—C1	1.784 (3)	C3—C8	1.508 (4)
N1—C7	1.141 (4)	C4—C5	1.396 (4)
N2—C4	1.383 (3)	C5—C6	1.379 (4)
N2—H2A	0.89 (3)	C5—H5	0.9500
N2—H2B	0.85 (4)	C6—H6	0.9500
C1—C6	1.384 (4)	C8—H8A	0.9800
C1—C2	1.384 (4)	C8—H8B	0.9800
C2—C3	1.387 (4)	C8—H8C	0.9800
C2—H2	0.9500		
			
C7—S1—C1	99.62 (12)	C5—C4—C3	119.5 (2)
C4—N2—H2A	116.2 (18)	C6—C5—C4	121.0 (2)
C4—N2—H2B	119 (2)	C6—C5—H5	119.5
H2A—N2—H2B	112 (3)	C4—C5—H5	119.5
C6—C1—C2	119.9 (2)	C5—C6—C1	119.6 (2)
C6—C1—S1	119.4 (2)	C5—C6—H6	120.2
C2—C1—S1	120.6 (2)	C1—C6—H6	120.2
C1—C2—C3	121.6 (2)	N1—C7—S1	178.8 (3)
C1—C2—H2	119.2	C3—C8—H8A	109.5
C3—C2—H2	119.2	C3—C8—H8B	109.5
C2—C3—C4	118.4 (2)	H8A—C8—H8B	109.5
C2—C3—C8	120.5 (2)	C3—C8—H8C	109.5
C4—C3—C8	121.2 (2)	H8A—C8—H8C	109.5
N2—C4—C5	119.5 (2)	H8B—C8—H8C	109.5
N2—C4—C3	121.0 (2)		
			
C7—S1—C1—C6	−96.9 (2)	C2—C3—C4—C5	0.8 (4)
C7—S1—C1—C2	86.6 (2)	C8—C3—C4—C5	−179.2 (2)
C6—C1—C2—C3	0.4 (4)	N2—C4—C5—C6	176.7 (3)
S1—C1—C2—C3	176.84 (18)	C3—C4—C5—C6	−0.4 (4)
C1—C2—C3—C4	−0.8 (4)	C4—C5—C6—C1	0.1 (4)
C1—C2—C3—C8	179.2 (2)	C2—C1—C6—C5	−0.1 (4)
C2—C3—C4—N2	−176.3 (2)	S1—C1—C6—C5	−176.5 (2)
C8—C3—C4—N2	3.7 (4)		

### 2-Methyl-4-thiocyanatoaniline Hydrogen-bond geometry (Å, º)

**Table:**

*D*—H···*A*	*D*—H	H···*A*	*D*···*A*	*D*—H···*A*
C8—H8*A*···N1^i^	0.98	2.61	3.482 (4)	148
C5—H5···N1^ii^	0.95	2.63	3.576 (3)	172
N2—H2*B*···N1^iii^	0.85 (4)	2.38 (4)	3.185 (4)	157 (3)

Symmetry codes: (i) −*x*+1/2, −*y*+1, *z*−1/2; (ii) −*x*+1, *y*+1/2, −*z*+3/2; (iii) −*x*+3/2, −*y*+1, *z*−1/2.
